# Sex Differences in the Level of Homocysteine in Alzheimer’s Disease and Parkinson’s Disease Patients: A Meta-Analysis

**DOI:** 10.3390/brainsci13010153

**Published:** 2023-01-15

**Authors:** V. Phu Nguyen, Andrila E. Collins, Jordan P. Hickey, Julia A. Pfeifer, Bettina E. Kalisch

**Affiliations:** Department of Biomedical Sciences and Collaborative Specialization in Neuroscience Program, University of Guelph, Guelph, ON N1G 2W1, Canada

**Keywords:** homocysteine, Alzheimer’s disease, Parkinson’s disease, sex differences, meta-analysis

## Abstract

Although recent studies suggest homocysteine (Hcy) is an independent risk factor for neurodegenerative disorders, little is known about sex differences in the levels of Hcy. In this study, we conducted a comparative meta-analysis to investigate sex differences in the levels of Hcy in both Alzheimer’s disease (AD) and Parkinson’s disease (PD) patients. Reports of Hcy stratified by sex in both AD and PD patients were obtained from electronic databases. From the initial 1595 records, 921 were assessed for eligibility, of which 16 sufficiently reported sex differences. Standardized mean difference (SMDs) using random effects together with tests of heterogeneity and quality assessment were applied in this meta-analysis. Data from 3082 diagnosed patients (1162 males and 1920 females) were included. There were statistically significant differences in the levels of Hcy between sexes in AD and PD patients, with an SMD of 0.291 [0.17, 0.41], *p* < 0.05, 95% CI, with higher Hcy levels detected in males. Subgroup comparisons did not find a statistically significant difference in the levels of Hcy between AD and PD patients. The overall risk of bias for the analyzed studies was low, with some moderate risk of bias across select domains. This meta-analysis determined that compared to females, males with either AD or PD have higher levels of Hcy. These findings suggest that Hcy could be a useful biomarker for predicting neurodegenerative diseases in males; however, further studies are needed to confirm the clinical utility of this suggestion.

## 1. Introduction

Alzheimer’s disease (AD) and Parkinson’s disease (PD) are chronic neurodegenerative disorders that involve the loss of neurons and synapses and the deposition of aberrant proteins [1,2]. AD, the most common neurodegenerative disorder diagnosed in older individuals, is characterized by the progressive loss of memory and cognitive functions, while PD, the second most diagnosed neurodegenerative disease, is primarily a movement disorder [3].

### 1.1. Alzheimer’s Disease

Common neuropathological hallmarks of AD include the deposition of the extracellular amyloid plaques and intracellular neurofibrillary tangles which compromise cellular pathways, leading to neuronal apoptosis and progressive changes in brain structure and function [1,4,5]. The neuronal loss in AD is non-reversible and primarily localized in the hippocampal and cortical regions of the brain [2]. A study carried out using a mouse model of AD determined that amyloid depositions correlated with a disruption in neuronal synaptic transmission, cellular toxicity, and elevated levels of oxidative stress [5]. The presence of oxidative stress in AD is further associated with the promotion of pathological mechanisms, such as the hyperphosphorylation of tau, leading to neurofibrillary tangle generation, as well as a disruption in the communication between neurons, further exacerbating the improper processing of amyloid beta, leading to the deposition of senile plaques [1,4,5]. Diagnosed cases of AD reveal significant clinical symptoms over time, such as poor judgment, loss of memory, impaired speech, and higher risks of comorbidity [4]. Although diagnosis is currently based on medical history and the results of laboratory tests, physical examination, mental status, and neuropsychological tests, researchers are continuing the search for biomarkers that would enable an earlier and more reliable diagnosis of AD [1].

### 1.2. Parkinson’s Disease

The main neuropathological features of PD include loss of dopaminergic neuronal cells in the substantia nigra and substantial deposition of intracellular proteins or Lewy bodies consisting of aggregates of α-synuclein proteins [6]. The etiology of the disease has been suggested to be due to mitochondria dysfunction, oxidative stress, the production of reactive oxygen species, and environmental toxins [7]. Accumulated increases in oxidative stress and free radicals contribute to neuron loss and lead to the misfolding and aggregation of proteins, such as α-synuclein [2,7]. An in vivo study showed that PD was associated with the infiltration of leukocytes that contributed to the degeneration of dopaminergic neurons [8]. As neurodegeneration progresses and the levels of dopamine decrease, diagnosed patients experience a broad spectrum of symptoms, including stiffness, tremor, difficulties with balance and coordination, slow movement, and potentially loss of movement [9]. Like AD, no specific laboratory test or biomarker has been identified that can confirm PD diagnosis. Instead, diagnosis is based on a patient’s medical history, symptoms, laboratory tests, and neurological and physical exam results [7].

### 1.3. Homocysteine

Homocysteine (Hcy) is a sulfur-containing amino acid metabolite produced by the trans-methylation of methionine [10,11]. Extensive research on the roles of Hcy has found that Hcy is an independent risk factor for various pathological conditions such as stroke, hypertension, and diabetes mellitus [11,12,13]. Hcy has been implicated in increased oxidative stress and neurodegeneration, and an increase in the levels of Hcy in blood is a marker for vitamin B12 deficiency [10,14]. Although the pathologies differ, high levels of Hcy have been associated with both AD and PD [4]. A meta-analysis of serum Hcy and dementia involving 8669 participants identified a positive relationship between hyperhomocysteinemia and AD [15]. In addition, Hcy was found to disturb DNA methylation and cause increased accumulation of beta-amyloid [13,16]. A clinical trial that compared 97 individuals diagnosed with PD to 66 non-PD individuals reported an increased concentration of Hcy in all 97 PD patients, which correlated with clinical symptoms such as decreased motor performance and cognitive decline [17]. Furthermore, elevations in Hcy also cause increased permeability of the blood-brain barrier and alter glycine, folate, and vitamin B12 metabolism, which disrupt the neuroprotective properties of these vitamins [10].

### 1.4. Sex Differences

Sex is one of the predominant and unmodifiable risk factors in the development of neurodegenerative disorders [18]. Females experience a two-fold increased risk of developing AD compared to males [18]. In contrast, the prevalence of diagnosed PD is much higher in males [18]. Despite sex differences in the incidence of AD and PD, little is known about sex differences in the levels of Hcy in these diseases [19]. Evidence supporting sex differences in the level of Hcy and in the prevalence of AD and PD suggests these differences could result from decreased sex hormones over the lifetime, exposure to toxins, and socioeconomic status (education, race, and age) [14,19]. In this meta-analysis, we examined the gender differences in the levels of Hcy in AD and PD and provide a comprehensive analysis of the available evidence on Hcy concentrations in both diseases for both genders.

## 2. Materials and Methods

### 2.1. Search Methodology

This meta-analysis was guided by the Preferred Reporting Items for Systematic Reviews and Meta-Analyses (PRISMA) guidelines. Articles were identified by searching Medline Ovid (1946-Present), Embase Ovid (1947-Present), Web of Science (1900-Present), PsycINFO (1806-Present), and Cochrane Database of Systematic Reviews and Cochrane Central Register of Controlled Trials (2000-present). Articles were reviewed by two independent raters and in the case of conflicts, final consensus was reached by a third rater.

### 2.2. Criteria for Inclusion/Exclusion

We included studies from English-language peer-reviewed journals that (1) reported Hcy levels by sex/gender (observational and experimental studies) and (2) included patients who were diagnosed with AD or PD (criteria were based on *Diagnostic and Statistical Manual of Mental Disorders*, DSM-5/American Psychiatric Association). There were no limitations on age and patients could have pharmacological and surgical interventions or co-morbidities. Pre-clinical trials and non-peer-reviewed studies were excluded.

### 2.3. Risk of Bias Assessment

Risk of bias was assessed based on the Newcastle Ottawa Scale and the Cochrane Collaboration’s risk of bias assessment items [20]. These included selection bias, performance bias, detection bias, attrition bias, reporting bias, and other sources of bias [20]. Each article was randomly assigned to and rated by 2 independent raters; discrepancies were resolved by a third rater.

### 2.4. Data Extraction and Statistical Analysis

Patients and study characteristics were recorded, including baseline levels of Hcy (mean ± standard deviation (SD)), age, study design, and type of diagnosis.

Standard mean differences (SMD) or Cohen’s d effect size and 95% confidence level (CI) were used in this meta-analysis in that Hcy concentration levels could be measured by different instruments. Random-effects model was applied to account for statistical heterogeneity as diversity across sample sizes and distribution of sexes among studies was expected [21]. The magnitude of SMD was interpreted as follows:

SMD of 0.2 represents a small effect;

SMD of 0.5 represents a medium effect;

SMD of 0.8 represents a large effect.

Measurement of heterogeneity based on chi-square test (Q) was included in forest plots [21]. To quantify inconsistencies among studies, the degree of heterogeneity was calculated using Chi^2^ statistic (I^2^) [21]. An interpretation of (I^2^) was as follows:

I^2^ < 60% is regarded as moderate

I^2^ from 50% to 90% is considered substantial

I^2^ from 75% to 100% is considerable

Evaluation of publication bias across studies was visually quantified using funnel plots and Egger’s test [21]. Lastly, subgroup analysis was performed for each disorder. All statistical analyses were conducted using statistical software for data science (STATA, version 16).

## 3. Results

### 3.1. Number of Studies Included

**Figure 1:**
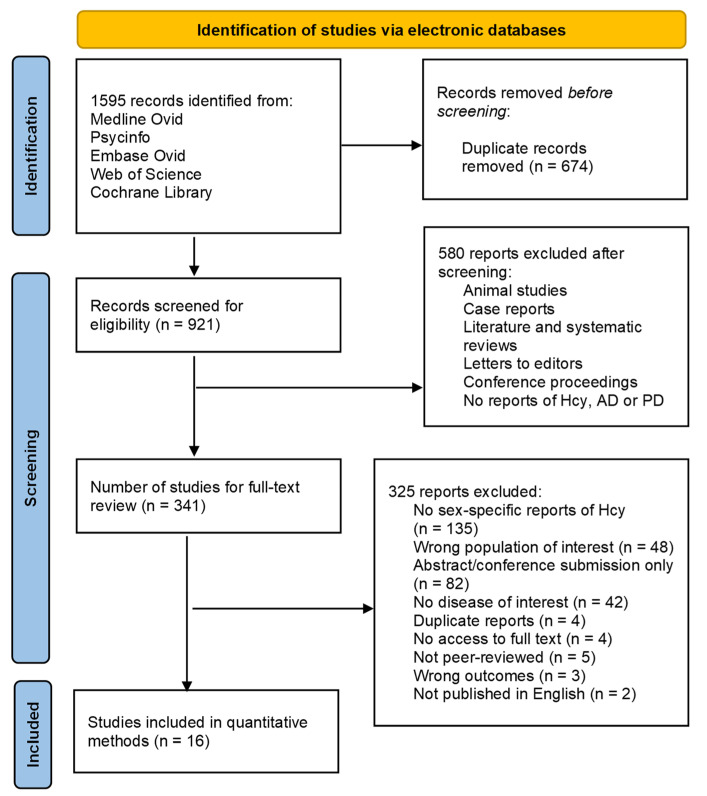
Inclusion and exclusion of studies guided by The Preferred Reporting for Systematic Reviews and Meta-Analyses (PRISMA).

The selection process of studies was guided by The Preferred Reporting Items for Systematic Reviews and Meta-analyses and is summarized in the flowchart shown in Figure 1. A search of studies via electronic databases (search terms indicated in Supplementary Table S1) resulted in 1595 records of articles, of which 921 articles were further assessed for eligibility with respect to study characteristics and Hcy outcomes. Following abstract and title screening, 341 articles underwent full-text review. Following a careful full-text review, a total of 16 studies were identified for inclusion in the meta-analysis. Most studies were excluded due to lack of reports of Hcy by gender or because they were reported as conference papers and abstracts. Sex-stratified Hcy levels were reported in 16 articles, of which eight papers focused on AD, seven papers examined patients with PD, and one paper investigated multiple neurodegenerative diseases (mild cognitive impairment, AD, and vascular dementia) [4,10,12,22,23,24,25,26,27,28,29,30,31,32,33,34]. A total of 3082 diagnosed patients (N = 1162 males) were included in the quantitative synthesis. Table 1 summarizes the characteristics of the 16 studies analyzed, including year of publication, country, diagnosis, diagnostic test, number of males and females, and mean age. Although some studies included data from individuals with other conditions, Table 1 only includes data for individuals diagnosed with either AD or PD.

### 3.2. Sex Differences in the Levels of Hcy in Both AD and PD Patients

**Figure 2:**
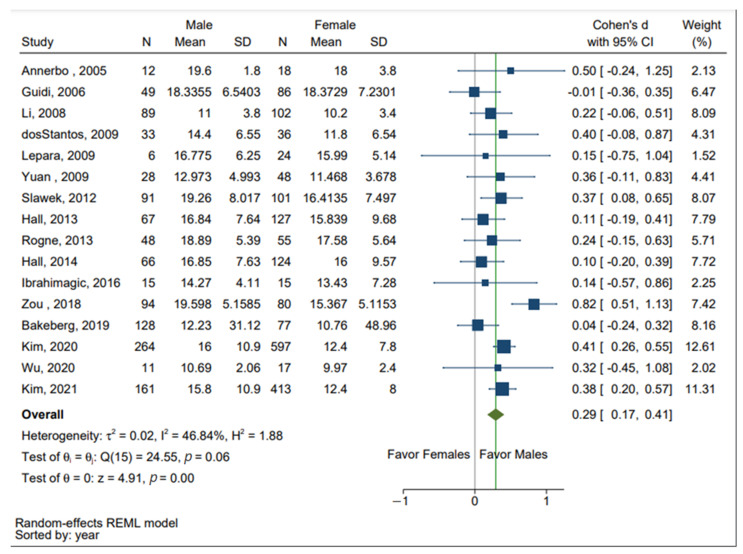
Forest plot displaying standardized mean differences (SMDs) or Cohen’s d in the concentration of Hcy in males (positive values) and females (negative values), 95% confidence intervals (CIs). The square symbols (■) represent the SMD in each study, with the size of the symbol being proportional to the precision of the estimate and the error bars indicating the 95% CI. The diamond symbol (♦) is the estimated overall effect. This plot is based on data presented in all 16 papers identified as eligible for this meta-analysis [4,10,12,22,23,24,25,26,27,28,29,30,31,32,33,34].

The results of the meta-analysis are presented in Figure 2. The Forest plot depicts significant gender difference in the concentration of Hcy in PD and AD patients combined. As indicated by the SMDs and overall effect size (green diamond shown in Figure 2), significantly higher levels of Hcy were detected in males. The overall effect size was Z score = 4.91, with an SMD of 0.291 [0.17, 0.41], *p* < 0.05, 95% CI. The test of heterogeneity using Chi^2^ statistic revealed moderate heterogeneity across studies, I^2^ = 46.84%, Q (2, 15) = 24.5, *p* > 0.05. Of the 16 studies, four studies (Slawek 2012, Zou 2018, Kim 2020, and Kim 2021) reported significant differences in the levels of Hcy stratified by sex [25,30,32,34].

### 3.3. Subgroup Analysis of AD and PD

**Figure 3:**
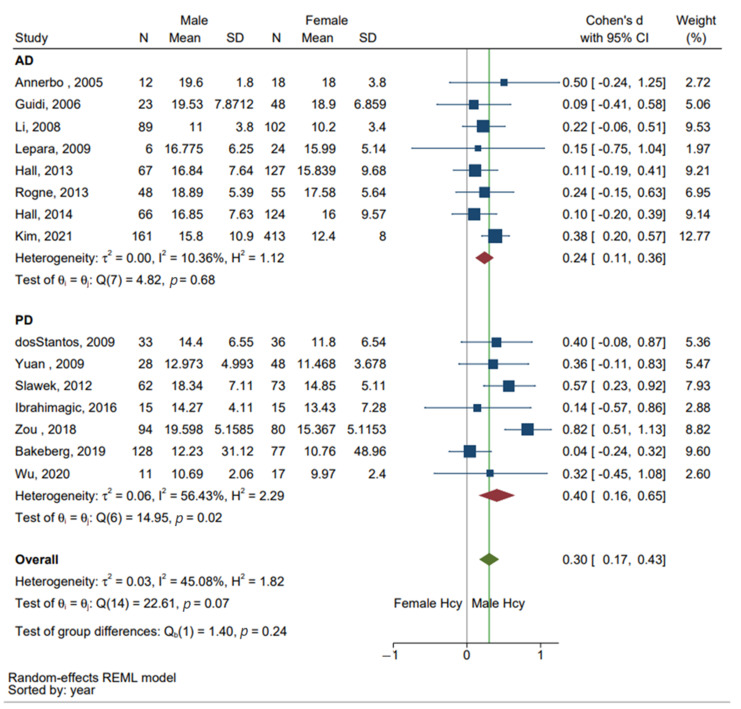
Subgroup analysis of Hcy levels in Alzheimer’s disease (AD) and Parkinson’s disease ( PD). A total of 15 studies were included in this analysis [4,10,12,22,23,24,25,26,27,28,29,30,31,32,33,34]. The Kim 2020 study was removed from subgroup analysis due to reports of total Hcy from multiple neurological disorders [32]. Both standard mean difference SMD (Cohen’s d) and 95% confidence interval (CI) are presented where Hcy levels from males are featured in positive values and Hcy levels from females are featured in negative values. The square symbols (■) represent the SMD in each study, with the size of the symbol being proportional to the precision of the estimate and the error bars indicating the 95% CI. The red diamond symbol (♦) is the estimated overall effect for each disease and the green diamond symbol (♦), the overall effect for both AD and PD.

A between-study subgroup analysis was performed to evaluate the sex differences in Hcy between individuals diagnosed with AD and PD. Figure 3 depicts SMD and overall effect size in this analysis. A total of eight studies reporting Hcy levels in patients with AD studies and seven studies of individuals diagnosed with PD were included in the quantitative analysis. The study by Kim, 2020 was removed from the subgroup analysis as the study included multiple disorders, with sex stratified Hcy levels in individuals with AD including those with and without cerebrovascular disease. No statistically significant difference in the levels of Hcy by disorder was found based on test of group differences Q (1) = 1.40, SMD: 0.3 [0.17, 0.43], *p* > 0.05. Overall heterogeneity across AD and PD remained moderate, I^2^ = 45.08%, Q (14) = 22.61, *p* > 0.05. However, subgroup analysis of the seven PD studies was found to have substantial heterogeneity, I^2^ = 56.43%, Q(6) = 4.82, *p* < 0.05.

### 3.4. Risk of Bias Assessment

**Figure 4:**
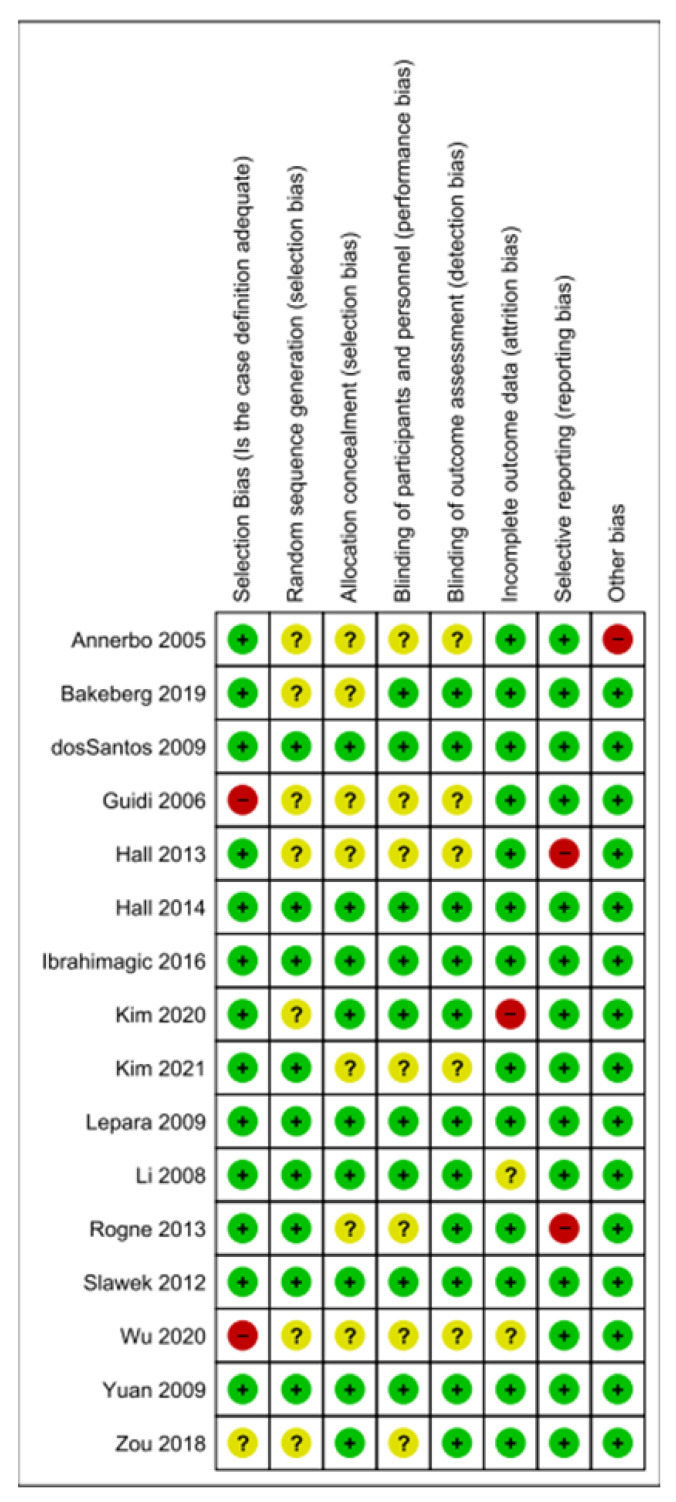
Summary of risk bias assessment for each study. Studies are indicated by the last name of the first author and year of publication [4,10,12,22,23,24,25,26,27,28,29,30,31,32,33,34]. Seven risk of bias domains were assessed, including selection bias, random sequence generation, allocation concealment, blinding of participants and personnel, blinding of outcome assessment, incomplete outcome data, and selective reporting; green = low risk of bias, yellow = unclear risk of bias, red = high risk of bias.

**Figure 5:**
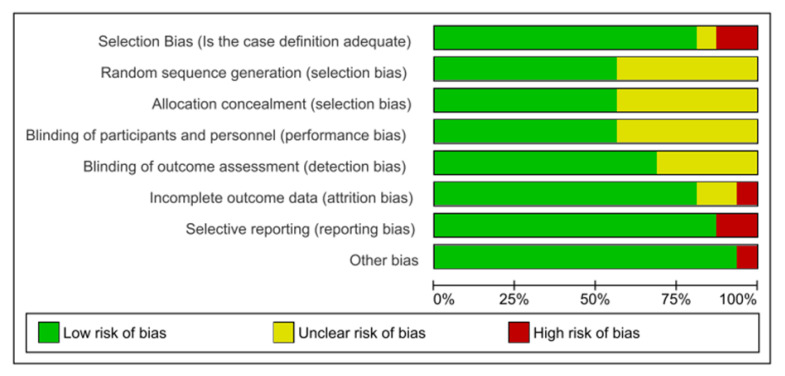
Overall summary of risk bias for each type of bias for all 16 studies; green = low risk of bias, yellow = unclear risk of bias, red = high risk of bias.

The risk of bias was assessed in each of the 16 included studies using the Newcastle Ottawa Scale and the Cochrane Collaboration’s risk of bias assessment items. Figure 4 represents a summary of the risk of bias in each of the individual studies, including selection bias, random sequence generation, allocation concealment, blinding of participants and personnel, blinding of outcome assessment, incomplete outcome data, and selective reporting. Figure 5 shows the overall proportion of bias assessed across all 16 studies. The category “other bias” includes any clear signs of bias not addressed in the domains indicated, as well as funding concerns or conflicts of interest.

We established that all papers had either a low or unclear risk of bias in four domains: random sequence generation, allocation concealment, blinding of participants, and blinding of outcome assessment. Since most studies were neither randomized controlled trials (RCT) nor interventional, there was a low likelihood of both performance and detection bias across studies. Two studies were determined to have a high risk of selection bias (Guidi 2006 and Wu 2020) [22,23], and two were found to have high reporting bias (Hall 2013 and Rogne 2013) [26,27]. However, no study included more than one source of high risk of bias. Out of the 16 papers included, we determined that six studies had a low risk of bias across all categories. As indicated in Figure 5, the overall risk of bias for all studies was low, with some moderate risk of bias across select domains. Evaluation of publication bias across studies was also visually quantified via funnel plot as indicated in Supplementary Figure S1 and Egger’s test, shown in Supplementary Figure S2.

## 4. Discussion

Neurodegenerative diseases are common pathologies seen in the elderly, resulting in significant poor health outcomes [1,2]. Previous studies have shown that elevated levels of Hcy are associated with increased risks of developing AD and PD [15,17]. This meta-analysis examined published articles and visually quantified previous results to investigate whether there was a significant sex difference in the level of Hcy in individuals diagnosed with AD and PD. Sixteen studies were found to meet the inclusion and exclusion criteria for meta-analysis and as shown in Figure 2, a significant sex difference in the level of Hcy across 3082 AD and PD patients (1162 male and 1920 female patients) was identified. A study conducted by Wang and colleagues that investigated Hcy levels in multiple neurological diseases found that male patients had Hcy levels that were 5 umol/L higher than the Hcy levels in female patients [35]. It was found that lifestyle and age had little effect on Hcy but sex served as the dominant factor contributing to the disparity in Hcy level [35].

A study published in 2020 investigating the levels of Hcy in 7872 healthy participants found that there was a significant difference in the levels of Hcy by sex for all age ranges from 20 to 80 years of age [36]. In the absence of neurological disorders such as AD and PD, physiological levels of Hcy were higher in males compared to their female counterparts [36]. Hcy levels in healthy individuals ranged from 5 to 15 umol/L [36]. The wide variation in the Hcy levels in individuals without neurological conditions was attributed to various factors, including age, differences in muscle mass, differential effects of hormones on the body, and different rates of Hcy formation [37]. Independent risk factors, such as smoking, and nutritional deficiencies, such as vitamin B12 or B6 deficiency, could also increase Hcy levels in healthy individuals [36].

In addition to an increased prevalence of AD in females, sex differences have also been reported for clinical symptoms, disease progression, and prognosis [reviewed in [18]. Several studies also examined the association between Hcy levels and dementia; however, the results were not always consistent and may be influenced by several factors, including sex. Annerbo and colleagues examined the connection between high Hcy levels and the development of AD in patients with mild cognitive impairment (MCI) and reported no correlation between Hcy levels and cognitive function, as assessed by Mini-Mental State Examination (MMSE) scores. Other studies reported inverse correlations between cognitive impairment, assessed by MMSE, and Hcy levels [10,23] and a lack of sex-specific differences in MMSE scores and Hcy levels [10,23,26,29]; however, when AD patients were separated based on symptoms or genetic risk factors, some differences were identified. In females, elevated serum Hcy levels correlated positively with neuropsychiatric symptoms as assessed by the Neuropsychiatric Inventory (NPI), hyperactivity, and affective symptoms [26]. In contrast, in males, Hcy levels were positively correlated with symptoms of psychosis [26]. When separated by apolipoprotein E (apoE) status, higher serum Hcy levels in females were a predictive factor for affective symptoms in carriers of the ε4 allele of ApoE and hyperactivity in non-ApoE-ε4 carriers, while no significant correlations between Hcy and symptoms in males were reported [28]. Although Kim et al. found overall higher levels of Hcy in males with AD, the number of APOE-ε4 alleles was significantly correlated with elevated Hcy levels in females and reduced Hcy levels in males [32]. Interestingly, the severity of medial temporal lobe atrophy was also correlated with elevated serum Hcy levels in addition to other factors such as female sex, low education, high risk of cardiovascular disease, and advanced age [32].

Sex differences in PD also include different clinical manifestations of the disease. The incidence ratio of developing PD for males versus females was found to vary from 1.37 to 3.7 [38]. In addition to sex differences in the prevalence of PD, clinical symptoms of the disorder were also reported to differ by sex in terms of severity and progressive development of the disorder [39]. For example, women were more likely to develop a tremor and postural instability while men were more likely to experience rigidity [2,3]. Both genetic and epigenetic factors could contribute to the underlying factors for the sex differences in clinical profiles of PD [19,31]. Bertogliat and colleagues reviewed multiple studies from in vitro cell culture models to the post-mortem analysis of human brains that have identified epigenetic dysregulation as a contributing factor in neurodegenerative diseases [40]. Studies of animal models have also identified sex differences in the epigenome during brain development that could contribute to the development of neurological conditions later in life [41]. Since hyperhomocysteinemia can induce epigenetic alterations and neurotoxic cascades, a better understanding of sex differences in Hcy levels in disease could provide insight into potential pathological consequences and sex-specific treatment strategies.

Based on the results of this meta-analysis and previous research into the different clinical presentations of neurodegenerative diseases by gender [31,42], we suggest that Hcy could be one of the factors that contribute to the severity of gender-specific differences in symptoms in neurodegenerative disorders, specifically PD. A 2017 study of sex-specific associations with cognitive impairment in PD found a positive association between elevated levels of Hcy and severe motor impairment [15]. It was confirmed that high levels of Hcy were highly predictive of increased scores in the Movement Disorder Society-Sponsored Revision of the Unified Parkinson’s Disease Rating Scale (MDS-UPDRS III) [35]. In addition to detecting significantly higher serum Hcy levels in males, Zou et al. identified a positive correlation between Hcy levels and Hoehn and Yahr scale staging, and a negative correlation between Hcy levels and MMSE scores in individuals living with PD who were also experiencing significant cognitive impairment [30]. These findings suggest high levels of serum Hcy may negatively influence motor and functional ability as well as cognitive function in this subset of PD patients. Bakerberg and colleagues reported that elevated serum Hcy levels in males, but not females, significantly correlated with higher MDS-UPDRS III, indicating elevations in Hcy levels are predictive of motor impairment in males but not females [31]. In contrast, elevated serum Hcy levels in females, but not males, were inversely correlated with Addenbrooke’s Cognitive Examination (ACE-R) scores, indicating elevations in Hcy levels in PD are predictive of cognitive impairment in females, but not males [31]. It is important to note that the findings discussed above are correlational and further studies, including longitudinal aging studies examining associations between serum Hcy levels and the development and progression of neurodegenerative diseases in males and females, are needed to confirm the clinical applications of Hcy.

Our study had limitations. First, most studies included in the quantitative analysis were retrospective and observational, which prevents the establishment of a causal relationship between the severity of the disorders and elevated levels of Hcy. In addition, sex differences in symptom severity were not reported in a sufficient number of the studies analyzed, and thus Hcy levels were not compared to the scores obtained for mental status or mobility. Second, certain medications and co-morbidity may have contributed to the different levels of Hcy; factors that were not explored in this study. For example, both cardiovascular and cerebrovascular diseases are associated with hyperhomocysteinemia [13,25]. Neuromodulation and neuropathology of both PD and AD are attributed to many factors, including nutrition, lifestyle, and therapeutic treatments. In this meta-analysis, we only studied sex differences. Future work should investigate both sex and age concomitantly to better our understanding of the associations of Hcy with aging and neurodegenerative disorders. Future research should also examine whether sex differences in Hcy levels are associated with the severity of specific symptoms or treatment outcomes. Though we were able to conduct a comparative analysis of Hcy by sex, sex differences in symptoms such as cognitive symptoms assessed by MMSE scores were not included in this analysis, as most studies did not report scores by sex.

## 5. Conclusions

The current study assessed sex differences in the levels of Hcy in patients with AD and PD. To our knowledge, this is the first meta-analysis reporting elevated Hcy levels in male patients with AD and PD. This supports the exploration of Hcy as a risk factor and potential biomarker for the development and progression of neurodegenerative diseases such as AD and PD. Although the results contribute to our understanding of sex differences in neurodegenerative conditions, additional preclinical and clinical investigations are required to further confirm the results of this study.

## Figures and Tables

**Table 1:** Characteristics of Studies Included in the Meta-analysis.

First Author	Year	Country	Diagnosis	Diagnostic Test	Sample Size F/M	Mean Age (Years) F/M *	Reference
Annerbo	2005	Sweden	AD	MMSE	18/12	69.2/65.6	4
Guidi	2006	Italy	AD	NINCDS-ADRDA	48/23	78	22
Li	2008	United States of America	AD	NINCDS-ADRDA	102/89	72.1/73.1	10
dosSantos	2009	Brazil	PD	UDPRS-motor score, HY staging and L-DOPA response	36/33	61.6	12
Lepara	2009	Bosnia and Herzegovina	AD	NINCDS-ADRDA	24/6	79.96	23
Yuan	2009	Taiwan	PD	HY staging	68/28	71.37	24
Slawek	2012	Poland	PD	UDPRS-motor score, HY staging	101/91	63.7	25
Hall	2013	United States of America	AD	NINCDS-ADRDA	127/67	78.31/75.17	26
Rogne	2013	Norway	AD	NINCDS-ADRDA	55/48	73.97/74.49	27
Hall	2014	United States of America	AD	NINCDS-ADRDA	124/66	78.57/75.43	28
Ibrahimagic	2016	Bosnia and Herzegovina	PD	tremor, bradykinesia, rigidity and postural abnormalities	15/15	62.73/65.6	29
Zou	2018	China	PD	UDPRS and HY staging	80/94	67.88	30
Bakeberg	2019	Australia	PD	UDPRS and HY staging	77/128	64	31
Kim	2020	South Korea	AD	NINCDS-ADRDA	597/264	75.3/73.2	32
Wu	2020	China	PD	MDS criteria	107/146	62/63.8	33
Kim	2021	South Korea	AD	NIA-AA	413/161	73.2	34

Studies sorted by year. * Age was not always separated by gender. Abbreviations: AD, Alzheimer’s disease; MDS, Movement Disorders Society; MMSE, Mini-Mental State Examination; NIA-AA, National Institute on Aging-Alzheimer’s Association criteria; NINCDS-ADRDA, National Institute of Neurological and Communicative Disorders and Stroke-Alzheimer’s Disease and Related Disorders Association (Alzheimer’s criteria); PD, Parkinson’s disease; UDPRS, Unified Parkinson’s Disease Rating Scale—motor score; HY, Hoen and Yahr.

## Data Availability

The authors declare that data supporting the findings of this study are available within the article or supplementary material.

## Supplementary Materials

The following supporting information can be downloaded at: https://www.mdpi.com/article/10.3390/brainsci13010153/s1, Table S1: Search Terms; Figure S1: Funnel Plot; Figure S2: Egger’s Test Results.
