# Supplementary material for: Sex Differences in the Level of Homocysteine in Alzheimer’s Disease and Parkinson’s Disease Patients: A Meta-Analysis
Source: Brain Sci. 2023 Jan 15;13(1):153. doi: 10.3390/brainsci13010153 (PMC9856546; doi:10.3390/brainsci13010153)
Supplement: Supplementary file 1 [file brainsci-13-00153-s001.zip › brainsci-2114058-supplementary.pdf]

Table S1: Search Terms

Search Terms
“sex*”, “gender*” “sex difference*” “gender difference*”, “sex characteristic*”, “gender characteristic*”, “dementia”, “alzheimer's”, “cognitive deficit?”, “Cognitive impairment”, “cognitive decline”, “delirium”, “homocystein$”, “homocystin$”, “hyperhomocysteinemia”, “hyperhomocysteinaemia”, “hyperhomocyst?in?emi$”

All search terms were performed with key words **.mp.** as multiple purposes for the title, abstract, subject heading, author keywords and other relevant information

Figure S1: Funnel plot evaluating publication bias across studies

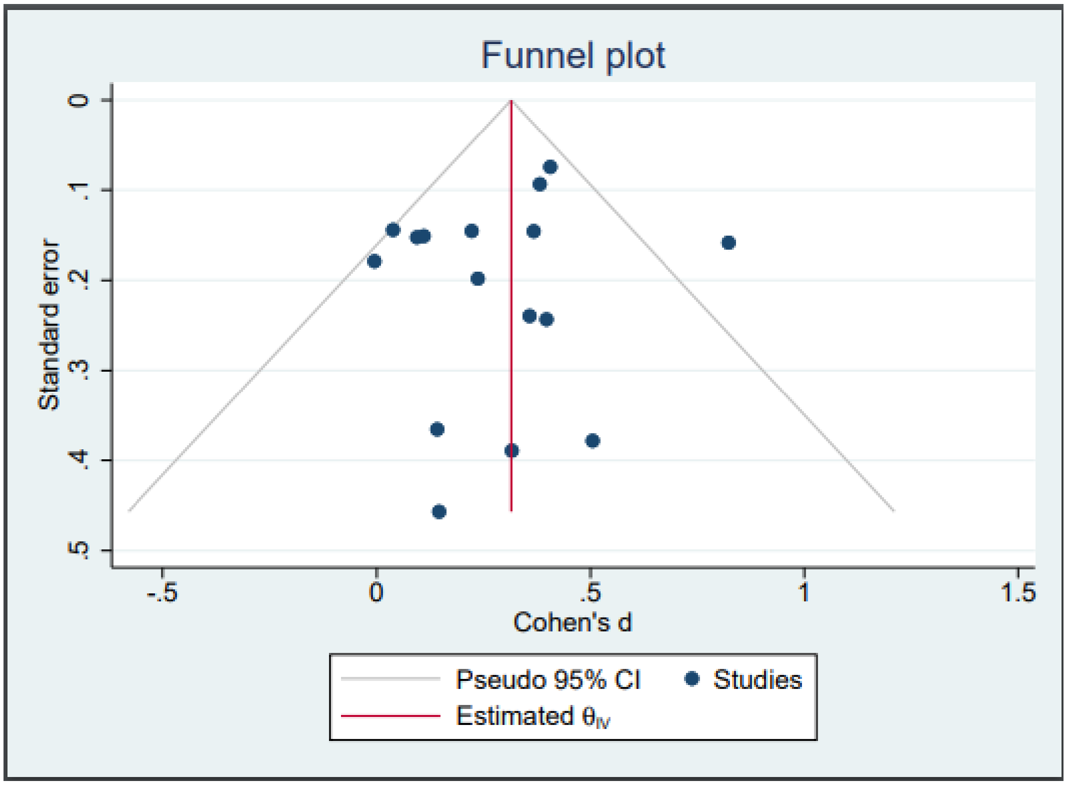

Figure S2: Egger's test results

```
H0: beta1 = 0; no small-study effects
            beta1 =      -0.23
      SE of beta1 =      0.742
                z =      -0.31
       Prob > |z| =     0.7600

.
```

Conclusion: No small- study effects, P =0.76 >0.05, low bias among studies
